# What Future for Protected Areas? Analysing the Mismatch between South Africa’s Pre-existing Protected areas System and the Declared vision in Contemporary Conservation Policy

**DOI:** 10.1007/s00267-024-02051-8

**Published:** 2024-09-23

**Authors:** R. C. Alberts, F. P. Retief, A. J. Bond, C. Roos, D. P. Cilliers

**Affiliations:** 1 Research Unit for Environmental Sciences and Management, North-West University, Potchefstroom, South Africa; 2 Protected Areas Research Group, North West University, Potchefstroom Campus, Potchefstroom, South Africa; 3 School of Environmental Sciences, University of East Anglia, Norwich, UK

**Keywords:** Conservation Policy, Protected Areas, Policy Orientations, Policy Implications, South Africa

## Abstract

Designation of protected areas has enjoyed global application as a means of biodiversity conservation for over 100 years. National conservation policy is essential as a means of protecting biodiversity, but is contingent on, amongst others, changing values and international drivers, and remains dynamic in many countries. As conservation policies evolve, the role of pre-existing protected areas within broader strategies for conservation can become unclear, with consequences both for the ability of the conservation policies to achieve their stated goals, and also for biodiversity outcomes within a nation. In order to map evolving inconsistencies between conservation policy and the role of protected areas within it, we develop a conceptual conservation policy framework synthesised from different policy orientations reported in the literature. Using South Africa as a case study, the conceptualisation is used to characterise the policy goals for protected areas in the recently adopted conservation policy, and the pre-existing protected areas system which remains on the statute books. The results indicate that the conceptual conservation policy framework can be used identify misalignment between policy and practice, and has enabled a mismatch to be identified between South Africa’s pre-existing protected areas system and its contemporary conservation policy, which suggests that the management of protected areas is likely to significantly change towards greater access and monetisation at the expense of their intrinsic value.

## Introduction

There is considerable debate around how we view individuals and societies in relation to nature and specifically in relation to conservation efforts and protected areas (Mace 2014; Colloff et al. 2017; Shume 2017; Kopnina et al. 2018; Sandbrook et al. 2019). These debates have been fuelled over time by, *inter alia*, changes in ecosystem structure driven by for example climate change and anthropogenic activities, changes in the perceived and literal functions and uses of nature, shifts in societal expectations and recognition of past injustices suffered by local communities in pursuit of conservation objectives, together with changes in the way in which society values and perceives nature (Colloff et al. 2017). Furthermore, there is a growing realisation that current funding models are inadequate to fully achieve conservation goals and outcomes and that there is limited understanding on the full value of conservation areas (Lindsay et al. 2020, Lessa et al. 2021). Mace (2014) illustrated these changes through her typology of conservation framings over time, divided into four eras: “Nature for itself” (pre-1970s); “Nature despite people” (1970’s to late 1990s); “Nature for people” (late 1990s to mid 2000s); and “People and nature” (mid 2000s to present day) (see also Sandbrook et al. 2019). In the “Nature for itself” framing the focus is on the prioritisation of wilderness and intact natural habitats, generally without people. This framing has its scientific underpinnings in wildlife ecology, natural history and theoretical ecology. “Nature despite people” focuses on threats to species and habitats from humans and on strategies to reverse or reduce them. “Nature for people” saw conservation thinking moving away from species and towards ecosystems as a focus for integrated management, with the goal of providing sustainable benefits for people in the form of ecosystem goods and services. The “People and nature” framing highlights a focus on people as part of ecosystems together with a focus on nature’s benefits and ecosystem services. This represents a shift towards greater recognition of the two-way, dynamic relationships between people and nature.

It is the changing worldviews underpinning these fluid debates that manifest in the literature as so-called “framings” or “orientations” (hereafter, “policy orientations”) (Mace 2014; Shume 2017). These orientations will by implication influence the direction of conservation policies and ultimately the objectives which they seek to achieve. The current policy orientation in any one jurisdiction is, therefore, a representation of the underlying guiding societal values at the time of adoption, often irrespective of the practical implications (See Tenbensel 2006).

Protected areas are recognised as being one of the most widespread conservation policy implementation instruments in pursuit of biodiversity protection (Watson et al. 2014). However, in many cases protected areas are historical designations based on societal values prevalent at the time. Of late, the efficacy and purpose of these areas have been questioned, with current debates being waged as to the suitability of protected areas in delivering conservation outcomes as espoused in broader conservation policy that reflects very different societal values. This is particularly evident within the context of the adoption of the Convention on Biological Diversity (CBD) (1992) which has as its objective the conservation of biological diversity and the sustainable use of its components (Convention on Biological Diversity, 1992: 4). The CBD ushered in a new era for protected areas, and how they were perceived to contribute to the growing commitment towards sustainable development (CBD, 1992:1). In keeping with the above, the CBD called for the integration of not only conservation, but also sustainable use, across relevant plans, programmes and polices (Convention on Biological Diversity, 1992: 6). Although recognising the important role played by protected areas in conservation, increasing calls were made for sustainable use and development within and adjacent to these areas, along the flow of ecosystem goods and services to serve society (Convention on Biological Diversity, 1992: 7). This arguably signalled a shift away from the original preservationist objectives of these areas. In response to the creation of policy aligned with the CBD, signatories would have been confronted with the potential mismatch between the historic and original concept of protected areas, and what these areas were now considered to be and to achieve.

In short – protected areas have been the preferred means of conservation long before the broader consideration of shifting societal values (as embedded in the CBD) have influenced the way we think about and view individuals and society in relation to conservation (Mayda 1969; Mace 2014; Colloff et al. 2017; Kopnina et al. 2018; Sandbrook et al. 2019). That is, emerging conservation policy needs to reflect current societal values and work out how pre-existing networks of protected areas can be integrated into politically-motivated conservation policies. Thus, despite a rich history of over 150 years, mandated and legislated protected area objectives can be misaligned with current societal views on the appropriate policy orientation to follow, or can simply be inconsistent by reflecting multiple policy orientations simultaneously, which jeopardises the achievement of the goals of any of the orientations (Pressey et al. 2015). The implications of such misalignment may result in protected area objectives having to be re-aligned with overarching policy objectives, potentially jeopardising their ability to “protect”. On the other hand, overarching policy objectives may not be fully achieved should protected areas not be re-aligned with the contemporary policy orientations that, politically, tend to follow contemporary societal values.

We recognise that questions about the effectiveness of conservation policy are necessarily value-based and contextual and we do not attempt to address them in this paper. Instead, our aim is to use identified policy orientations as a framework within which the alignment between existing protected areas policies, and contemporary conservation policy in a country can be evaluated. South Africa is used as a case country for three reasons: 1) protected areas date back 130 years; 2) the country has recently drafted its first national conservation policy making it an opportune time to evaluate; 3) from a pragmatic perspective, the authors have expertise related to conservation in the country and access to relevant stakeholders. That is, we aim to develop a tool for identifying misalignment between conservation policy aspirations and management practices given that such misalignment will necessarily preclude achievement of conservation policy objectives (and therefore, by definition, be ineffective).

This aim is broken down into three sequential objectives:

1. To develop a conceptual framework from literature for testing policy orientation alignment of conservation policies.
2. To test the utility of the conceptual framework using south Africa as a case country. This is achieved by analysing the alignment between the country’s existing legislated protected areas objectives against the stated visions for the same protected areas in contemporary conservation policy in South Africa.
3. Determine the possible implications of the findings on South Africa’s existing protected area system.

The developed conceptual framework has broader application as it provides a means of examining whether policy implementation instruments (i.e., the management of protected areas and other conservation areas) are aligned with current conservation policy orientations in any jurisdictional setting.

The next section sets out the methodological approach for the research. This is followed by Sections 3, 4 and 5, which present the findings for each of the three objectives in turn. Section 6 sets out the conclusions which focus on the utility and wider relevance of the conceptual framework, as well as indicating some broader lessons for policy makers (including those outside South Africa) that have emerged from the analysis.

## Methods

### Developing the Conceptual Framework

The difficulty when considering conservation policy, and specifically protected areas as policy implementation instruments, is not the scarcity of literature, but rather the surfeit of literature related directly or indirectly to the topic. The approach taken was to continue the literature review until theoretical saturation was achieved in line with the thinking of Strauss and Corbin (1998) and Hacking and Guthrie (2008). Keywords used (in various combinations) included ‘protected areas’ AND ‘conservation’ AND ‘policy’ AND ‘implementation’, AND ‘debates’, AND ‘orientations’, AND ‘objectives’ AND ‘dimensions’ AND ‘positions’. We conducted our search through the academic databases: Scopus and Google Scholar up to the end of December 2023. Our research does not attempt to systematically provide a complete overview of all literature related to conservation policy, but rather to identify dominant policy dimensions evident in the conservation discourse. The method used to develop the conceptualisation followed the approach of Jabareen (2009) and involved applying the following steps to the literature:

1. Identifying and naming concepts related to conservation policy;
2. Categorising the concepts;
3. Integrating the concepts; and
4. Synthesis, re-synthesis, and making it all make sense.

### Application of the Conceptual Framework

In pursuit of Objective 2 and using South Africa as a case country, relevant legal text and recently adopted conservation policy text relating to existing protected areas are evaluated against the conceptual framework by means of qualitative analysis adapted from Zhang and Wildemuth (2009) (see also Macura et al 2019). The use of a single case in the testing of the utility of the conceptual framework is supported by Yin (2012) and Flyvbjerg (2011). In order to test the utility of the framework, firstly, the relevant texts were searched using the keywords “protected area”. This delivered the sections of the documents dealing directly with conservation policy goals involving protected areas, or with the implementation objectives of protected areas. It must be noted that the policy document specifically does create some confusion in that it uses the terms “protected area” and “conservation areas.”^1^ Only those provisions which deal specifically and directly with protected areas were indexed and considered for analysis. Secondly, in pursuit of the aim, the texts dealing specifically with protected areas is read, and deductive coding (after Bryman 2016) is used to assign the text to the differing policy orientations as conceptualised in Fig. 2 (with triangulation by means of an expert workshop involving six academics). Text can often be ambiguous, leading to complications for deductive coding. Qualitative content analysis therefore allows the researcher to assign a unit of text, in this case the policy statement, to more than one theme (policy orientations) (See Tesch 1990). Thus, where specific policy text could be assigned to more than one of the policy orientations, all the relevant orientations are indicated as being present.

The results as discussed in Section 4, below, provide for an assessment of the alignment of the reference to protected areas in the new policy document with overarching policy orientations, whilst furthermore allowing for the assessment of the alignment of the existing policy implementation instrument on protected areas, with overarching policy orientations.

## Objective 1: Develop a Conceptual Framework from Literature for Testing Policy Orientation Alignment of Conservation Peerolicies

**Fig. 1:**
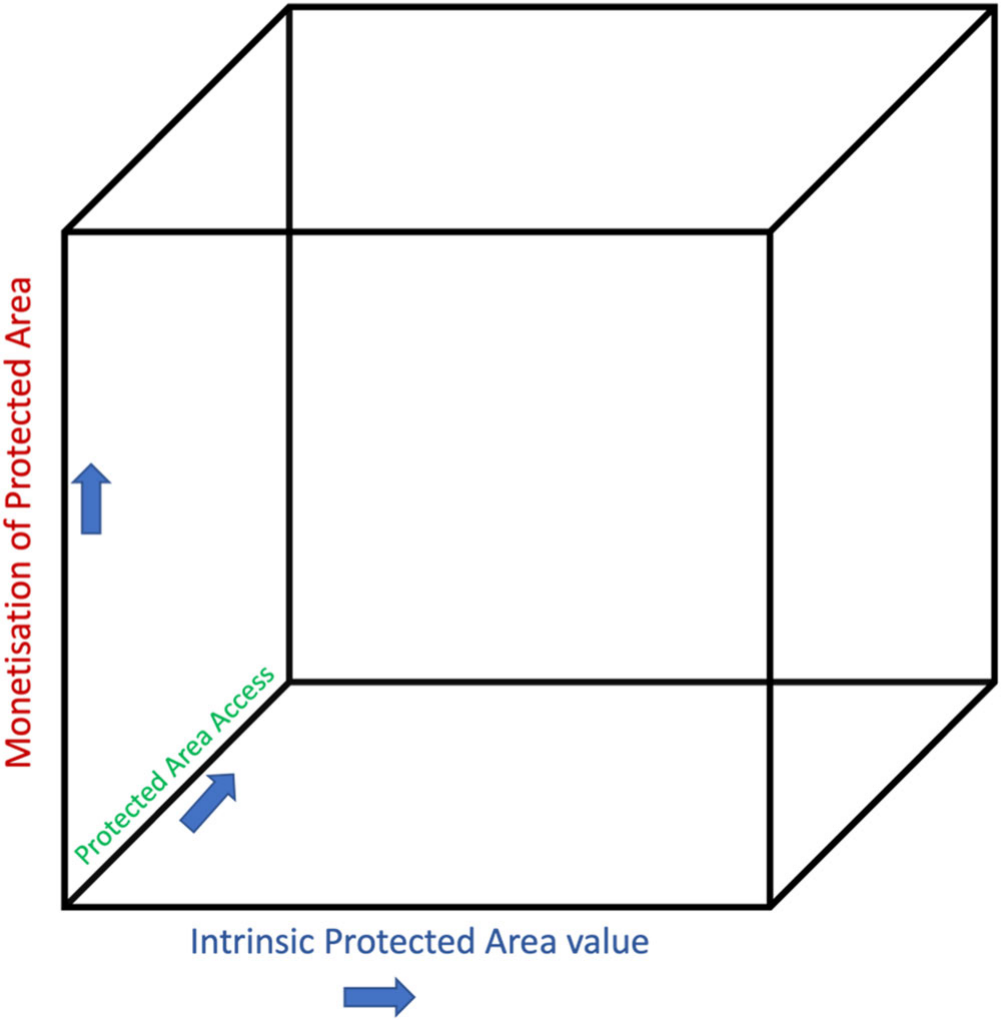
Three axes forming the basis for the conceptual framework
- *The level of access to the protected area*: A further central theme in the conservation and specifically protected area discourse relates to access. The spectrum extends from strictly protected areas with little to no human access to resources, through to areas with high levels of access to resources by communities (See Fig. 1, z-axis) (Rolston 1996, 1998; Siurua 2006; Holmes 2007; Coad et al. 2008; Tanner et al. 2010; Mascia and Pailler 2011; Holmes and Cavanagh 2016, Cundill et al. 2017, De Vos et al. 2018). Policymakers face the challenge of having to consider inclusive access while maintaining ecosystem integrity, highlighting potential tensions between limited access and no access and conservation thinking that integrates human wellbeing with ecological preservation.
- *The level of protected area monetisation:* The third theme identified relates to the extent to which markets are used to monetise protected areas, their benefits and the individual components within these areas on a spectrum from no monetisation to full monetisation (See Fig. 1, y-axis) (Stolton et al. 2008, 2015; Fletcher 2010; Brockington et al. 2012; Crist 2015; Holmes et al. 2017; Lessa et al. 2021; Retief et al. 2022). The discourse on monetisation reflects a deeper understanding of how economic incentives can be aligned with conservation goals, leaving policymakers to determine the extent to which protected areas are to be monetised so that they may contribute towards socio-economic development.

Based on the theoretical debates and discussion embedded in the literature identified during the literature review, analysis of concepts reveals that there are three underlying spectra which influence conservation policy debate, each spanning polar opposites. We argue that any considerations of the societal legitimacy of conservation policy influencing protected areas will have to take cognisance of, and deliberate as to, where on the following spectra a particular policy is located. These are namely:

- *The intrinsic value of the protected area*: The traditional divide between anthropocentrism and ecocentrism has long been a central theme within the conservation discourse (Pinchot 1910; Baxter 1974; Taylor 1981; Regan 1983; Wildes 1995; Agar 2001; Singer 2011; Purdy 2013; Shume 2017; Washington et al. 2017; Kopnina et al. 2018). This spectrum underscores the necessity of considering ‘value’ along a continuum that reflects the dynamic interplay between conserving nature for its own sake and for the benefits it provides to humanity (See Fig. 1, x-axis). This intrinsic value debate plays out in the answer to the fundamental question: “Why is this area being protected?” Policy makers will, therefore, have to consider whether protected areas and the components therein are to be conserved for their own sake, in and of themselves, or whether their protection is beholden to a utilitarian value for humans, or some compromise between the two.

Policy makers are thus inadvertently confronted with these spectra and forced to adopt a position along each. The point of intersection along the relevant spectra of intrinsic value, access, and monetisation, explains the conservation framing of any given policy and provides clues about both the conservation outcomes that might be expected as well as the potential societal legitimacy in a given context. These three spectra are depicted in Fig. 1 below.

To highlight currently advocated conservation worldviews within this conceptual framework, the Future of Conservation (FoC) survey (Future of Conservation, 2022), ostensibly driven by Sandbrook et al. (2019), which identifies four main orientations for conservation policy worldwide, was used as the benchmark for globally advocated policy orientations.

**Fig. 2:**
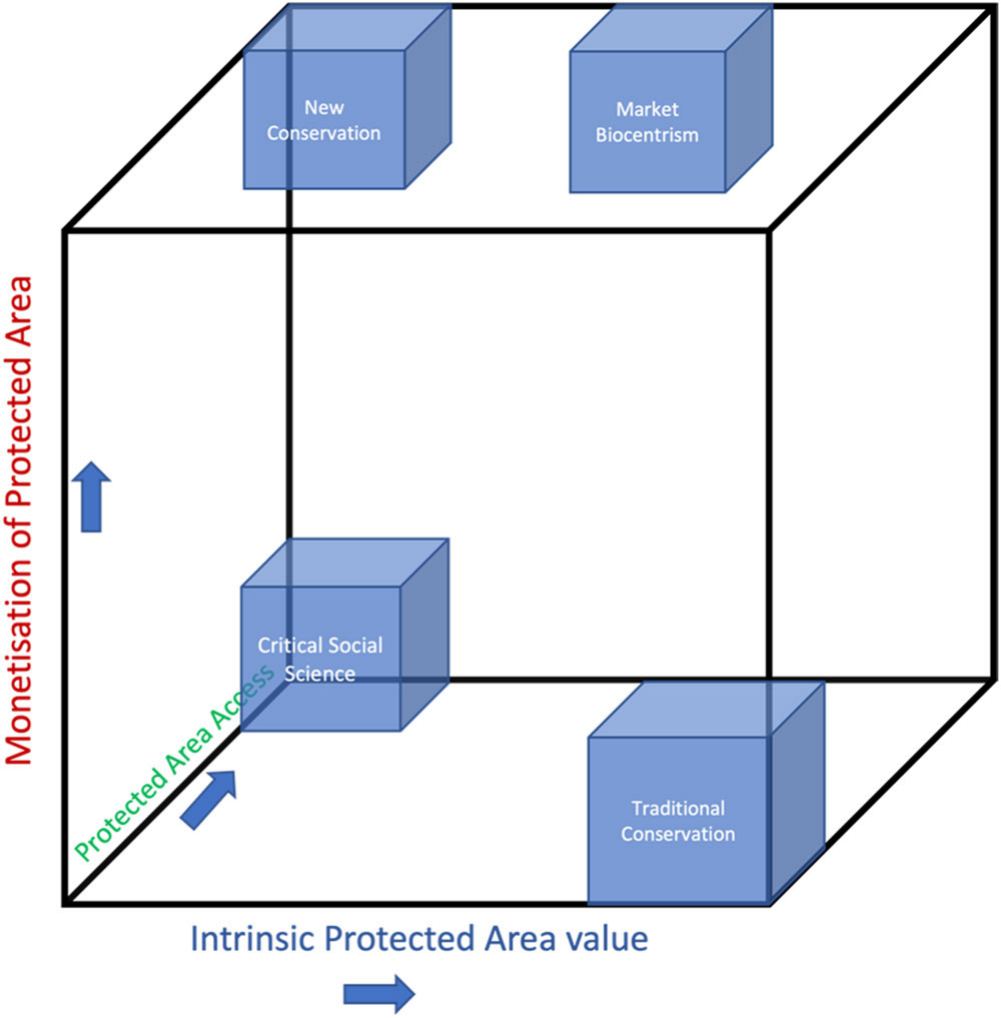
A conceptualisation of conservation policy orientations along the spectra of intrinsic value, monetisation and access

The four FoC orientations are summarised below and then placed in the conceptual framework, in Fig. 2, to illustrate their relative positioning on the three axes.

### Traditional Conservation

Traditional Conservation tends towards ecocentrism and aligns with Shume’s (2017) categorisation of ‘us in nature’ or what Mace (2014) refers to as ‘nature for itself’ and ‘nature despite people’. It is, furthermore, characterised by high intrinsic value of protected areas, low regard for the monetisation of these area, with limited or no access to protected areas. This policy orientation may be seen as idealistic, claiming that policy should be evaluated with a particular vision of “how things should be”, whilst ascribing to holistic views which are grounded in the belief that protected areas are not simply a collection of parts, but rather complex wholes from which emergent properties arise when the parts interact (Shume 2017).

Key concepts and themes underlying this orientation against which policy may be analysed are listed in Box 1.

Box 1 The Characteristics of the Traditional Conservation Orientation Intrinsic value • low monetisation • no/restricted access • holistic approaches to PA management • idealism • species focus • importance of wilderness as a concept • strong reliance on traditional protected areas for conservation • intrinsic value of protected areas is evident.

Source: Roe 2008; Kariva and Marvier 2012; Soulé 2013; Doak et al. 2014; Mace 2014; Wuerthner et al. 2015; Holmes et al. 2017; McKenzie 2017; Shume 2017; Kopnina et al. 2018; Bhola et al. 2021

### New Conservation

New conservation aligns with Mace’s (2014) framings of ‘nature for people’ and ‘people and nature’, as well as what Shume (2017) refers to as ‘technocentrism’ (us over nature). In essence a technocentric policy orientation states that humans hold the power and responsibility to manage protected areas to ensure sufficient natural resources from such areas are produced and conserved to meet current and future socio-economic demands (Shume 2017). This orientation is characterised by adopting an instrumental value for protected areas, relying heavily on protected area monetisation and by pursuing access to resources.

This policy orientation may be critiqued for adopting an unexamined optimism that science, markets, and engineering will generate solutions to control and propagate resources from and around protected areas sufficiently so as to meet ever increasing socio-economic demands. Our literature review records the earliest discussion of this ‘new conservation’ policy orientation as 1965 (Johnson 1965; O’Callaghan 1967; Mayda 1969).

Key concepts and themes underlying this orientation against which policy may be analysed are listed in Box 2.

Box 2 The Characteristics of the New Conservation Orientation High monetisation of protected areas • high access to resources in protected areas • pragmatic management views • reductionist views of protected areas • protected areas are valued for their instrumental value • win-win philosophy whereby protected areas can conserve nature and benefit socio economic aspirations • emphasis on community based natural resource management • sustainable use is a favoured concept, growth and investment of infrastructure within and around protected areas • economic value of protected areas is highlighted • increased focus on ecosystem services • reliance on resource and environmental economics.

Source: Johnson 1965; O’Callaghan 1967; Mayda 1969; Hulme and Murphee 1999; Brown 2003; Hall and Frost 2009; Miller et al. 2011; Minteer and Miller 2011; Karieva and Marvier 2012; Marvier 2012, 2014; Soulé 2013, 2014; Mace 2014; Petriello and Wallen 2015; Batavia and Nelson 2017; Holmes et al. 2017. Shume 2017; Kopnina et al. 2018

### Critical Social Science

In the ‘critical social science’ orientation the impacts of conservation on human well-being should be at the forefront of the conservation debate. This entails being critical of conservation activities that can have negative effects on people, such as creating protected areas that explicitly exclude access. It is also critical of a ‘nature-for-nature’s sake’ rationale for conservation and the use of natural science within conservation, rather aligning with the ‘nature for people’ framing (Mace 2014). This policy orientation aligns with what Shume (2017) calls ‘egocentrism’ or ‘us versus nature’ (see also Herman et al. 2015) and embraces a win/lose dichotomy, where protected areas are seen as being a means to fulfilment of human needs and wants which are prioritised over all other considerations. Critical social science scholars claim that protected areas as traditionally conceived are a pastime of the ‘elites’, seeing it as a colonialist residue of Western imperialism (Butler 2015). ‘Critical social science’ orientations are arguably grounded in a neo-Marxist world view which pursues the dominance of nature towards the benefit of humankind alone (Holmes and Cavanagh 2016; Kopnina et al. 2018). These narratives are underscored by the overall goal of improving human well-being, with the needs of humans prioritised.

Key concepts and themes underlying this orientation against which policy may be analysed are listed in Box 3.

Box 3 The Characteristics of the Critical Social Science Orientation Instrumental Value of protected areas is highlighted • low reliance on monetisation of protected areas • high access to resources within protected areas • a reductionist view of protected areas, an overly pragmatic approach to protected area management • a highly anthropocentric view of protected areas • sharp focus on the negative impacts of protected areas on the wellbeing of vulnerable and previously disadvantaged groups • sceptical views of markets and business • increased focus on past redress of impacts from protected area establishment and management • humans vs protected areas narrative is central • a win/lose dichotomy is argued, namely either people or protected areas stand to benefit, not both • people vs parks is a central theme • the pursuit and fulfilment of human needs is paramount • increased critique of protected areas as causing negative side effects on humans • protected areas should primarily improve human wellbeing • traditional protected areas are provenance of the elite neo liberal classes • favourable of a neo Marxist world view • critical of what is considered to be neo-protectionism.

Source: Mace 2014; Brockington and Wilkie 2015; Butler 2015; Crist 2015; Holmes et al. 2017; Holmes and Cavanagh 2016; Shume 2017; Kopnina et al. 2018.

### Market Biocentrism

Literature dealing with market ecocentrism is scant and some contend that the term market ecocentrism is an oxymoron, in that it attempts to merge the concepts of biocentrism with those of neoliberalism in an effort to pursue “ecologically enlightened self-interest” (Kopnina et al. 2018). The FoC survey (Future of Conservation 2022) cites one example of market ecocentrism, namely the recent Nature Needs Half movement (as well as the closely related Half-Earth movement) (Wilson 2016).

Market biocentrism is more difficult to characterise. Policy aligned with this orientation will recognise both the intrinsic and instrumental value of protected areas. It will pursue high degrees of monetisation which may be leveraged to further conserve protected areas. This will by implication necessitate high levels of access to resources in certain areas. It intertwines intrinsic value with market-based approaches and relies on markets to conserve protected areas. The central premise of this orientation is that ecological systems will prove to be resilient if key thresholds are not exceeded. The focus is thus not on policy towards human optimisation, but rather the pursuit of natural resilience.

Key concepts and themes underlying this orientation against which policy may be analysed are listed in Box 4.

Box 4 The Characteristics of the Market Biocentrism Orientation Tolerant of intrinsic and instrumental value views in relation to protected areas • high monetisation of certain protected areas in favour of conserving others • high access to resources within certain protected areas in order to conserve others • a more holistic view of protected areas • a reasonable degree of pragmatism with regard to protected area management • intertwines intrinsic value with markets based approaches • strong reliance on markets and capital to conserve protected areas • underlying belief that systems are resilient if key thresholds are not exceeded • management is not focused on human wellbeing and optimisation but rather nature’s resilience.

Source: Wilson 2016; Kopnina et al. 2018; Future of Conservation 2022.

## Objective 2: To Test the Utility of the Conceptual Framework by Analysing the Alignment between the Country’s Existing Legislated Protected Areas Policy Against the Stated Visions for the Same Protected Areas in Contemporary Conservation Policy in South Africa

South Africa’s protected areas system dates to the turn of the 19th century, with the proclamation of the first (colonial) protected area in Africa in 1894: the Pongola Nature Reserve. In 1926, the national government established the National Parks Board through the National Parks Act which together with numerous provincial ordinances saw the formation of different types of protected areas across the country, including, national parks, provincial parks, municipal reserves, and private nature reserves (Goosen and Blackmore 2019). Legislation regulating protected areas was adopted and the current protected areas system is centred around the National Environmental Management Protected Areas Act (NEMPAA) (RSA 2003) together with related and ancillary legislation at the national and provincial level. The NEMPAA makes provision for the declaration of different types of protected area, cascading from strictly protected to least protected (see Table 1). It sets out the objectives of protected areas (see also Supplementary Table 1). To date, South Africa has 1506 protected areas spanning the range of different types afforded by the NEMPAA, comprising approximately 9.9% of its terrestrial area, and 41 marine protected areas comprising 5% of the coastal and marine areas around its coast.

**Table 1:** Analysis of existing protected areas against policy orientations

Types of Protected Areas as set out in the National Environmental Management Protected Areas Act 57 of 2003	Policy Orientations Identified in Legislated Objectives
General purpose of protected areas Section 17	Traditional Conservation New conservation Market Biocentrism
Special Nature Reserves Section 18(2)	Traditional Conservation
National Parks Section 20(2)	Traditional Conservation New conservation
National Park Wilderness Area Section 22(2)	Traditional Conservation
Nature Reserves Section 23(2)	Traditional Conservation New conservation
Nature Reserve Wilderness Areas Section 26(2)	Traditional Conservation
Protected Environment Section 28(2)	Traditional Conservation New conservation

The legislated purpose and objectives of special nature reserves, national parks, nature reserves, wilderness areas and protected environments, as currently contained within legislation, were analysed against the conceptual framework as set out in Section 2.2 above. For the purposes of this research, marine protected areas were not considered in the analysis given the unique contextual factors differentiating them from terrestrial protected areas. The result of the analyses of the existing protected areas as policy implementation instruments against policy orientations are summarised in Table 1 (with a more detailed breakdown of specific paragraph coding set out in Supplementary Table 1).

**Fig. 3:**
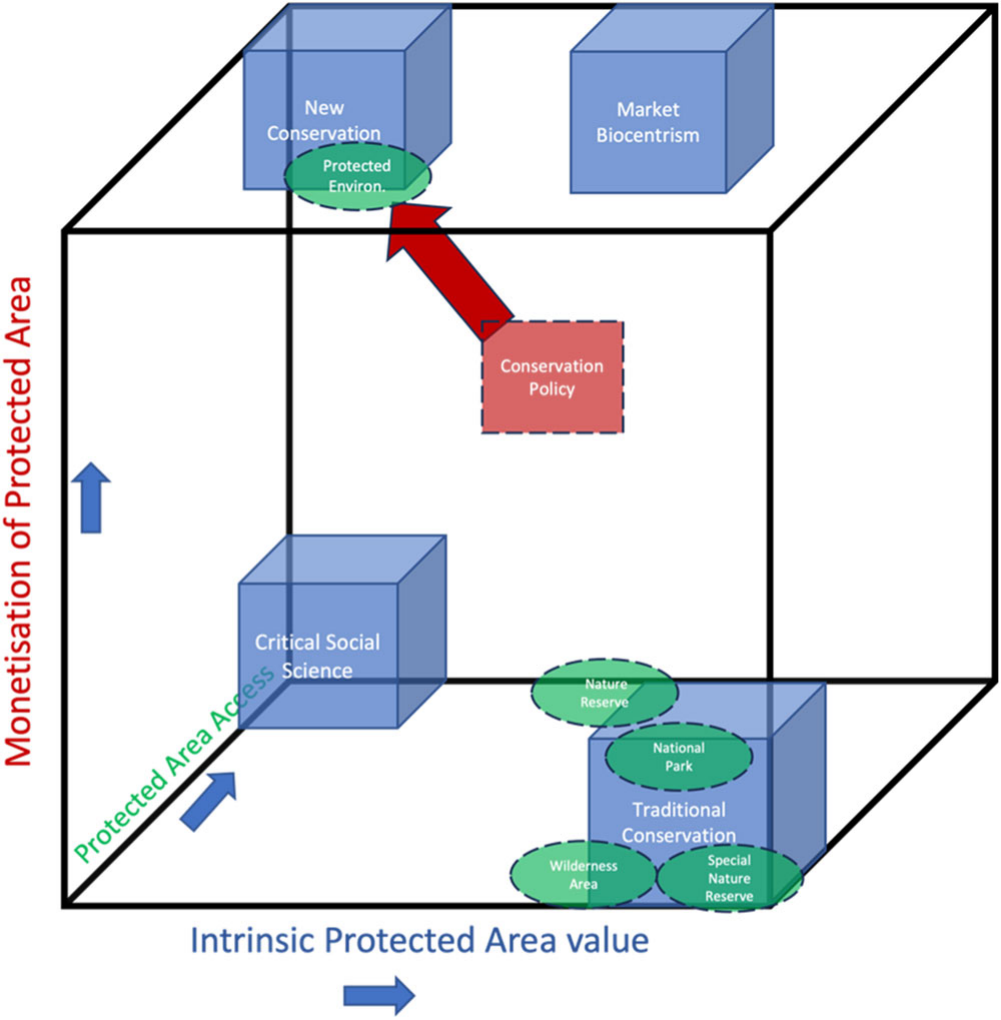
Policy tensions between new direction and current instruments

It is evident from the above analyses, that the overall orientation of the existing protected areas in the country, is predominantly traditional conservation focused (see Fig. 3 below). The only exception being protected environments, which incorporate to a greater extent objectives aligned with the new conservation orientation (Fig. 3). Although certain objectives of the analysed protected areas align with the new conservation orientation it cannot be said to be the overarching orientation in respect of the countries existing protected areas.

South Africa acceded to the CBD in November 1995 and subsequently mandated a new protected areas system within the country founded on a draft policy on Biodiversity and Conservation published in 1997 (RSA 1997). This remained an unpublished draft until a new draft policy on the Conservation and Sustainable Use of South Africa’s Biodiversity was published for comment on July 8th 2022 (RSA 2022) in an attempt to finally formalise conservation and biodiversity policy. The first draft was subsequently superseded by a second draft of the policy published for comment on October 28th 2022, with the final policy being adopted on 14 June 2023 as the “White Paper on the Conservation and Sustainable Use of South Africa’s Biodiversity” (hereafter the White Paper) (RSA 2023).

The newly adopted White Paper is an overarching policy that is aimed at addressing, *inter alia*, the lack of consensus among stakeholders on how to pursue conservation (RSA 2023: 5). The White Paper at face value espouses new conservation thinking, given that at a strategic level the policy aims to achieve policy certainty and a strong policy base for biodiversity conservation, whilst identifying protected areas “*as mechanisms to contribute strongly to ecologically sustainable rural development*” (RSA 2023:5). The above is contextualised against the vison of the newly adopted White Paper which reads as follows: “*A society living in harmony with nature, where biodiversity conservation and sustainable use is transformed, ensuring improved benefits from healthy ecosystems, that are fairly and equitably shared*” (RSA 2023:5). The policy sets out four goals (see Box 5), each with unique objectives, expected outputs and expected outcomes.

The four goals together with their unique objectives, expected outputs and expected outcomes were analysed using the method set out under Section 2.2 above. The results in the form of the analysed text of the White Paper together with the assigned policy orientations are illustrated in Table 2 and Fig. 3 (Supplementary Table 2 provides a more detailed breakdown of the coding of specific paragraphs of the White Paper against the four policy orientations).

**Table 2:** Analysis of relevant White Paper provisions against the policy orientations

Protected Areas Relevant Policy Goals	Protected Areas Relevant Policy Objective	Policy Orientation
Goal 1: Enhanced biodiversity conservation: All biological diversity and its components conserved.	1.1 Expand a representative system of protected and conservation areas that are effectively and efficiently managed	Traditional Conservation
	1.2 Better integrate conservation areas into broader ecological and social land- and seascapes	New Conservation
Goal 4: Transformed biodiversity conservation and sustainable use: Effect is given to the environmental right as contained in Section 24 of the Constitution which facilitates redress and promotes transformation.	4.2 Position protected and conservation areas as catalysts of inclusive socio-economic development.	New Conservation
	4.4 Promote participation and influence of designated groups (PDIs, youth, women and people with disabilities) in biodiversity conservation and sustainable use.	New Conservation Critical Social Science

The incorporation of, and orientation towards, new conservation thinking in South Africa’s new conservation policy in relation to protected areas is to be expected as it is an increasingly popular orientation among academics and policymakers, which dovetails with tactical shifts in the mission statements of many conservation organisations (Doak et al. 2014). This highlights what Terborgh and Peres (2017) describe as arguably a central challenge faced by conservationists, namely the clarification of the distinction between ecosystem services and biodiversity protection in the public mind, as it is becoming increasingly evident that conservation is operating in what Chapron and Lopez-Bao (2019) describe as an ethical frame whereby wild plant and animal species must first and foremost benefit human communities, and by implication become unacceptable if they impose a burden on people. The economic focus of new conservation may serve as the main objection of those wary or sceptical of this orientation, that is, stakeholders that align with other policy orientations.

The shift towards new conservation thinking for protected areas at the policy level, dictates a move away from the traditional policy orientation in which the majority of South Africa’s protected areas are rooted, namely that of traditional conservation (See Fig. 3). Already there have been calls for South Africa’s protected areas to reimagine their roles and to adapt beyond non-consumptive wildlife uses (Clements et al. 2022 see also Coetzee et al. 2022). The existing and historic legal (and arguably policy) framework within which these protected areas are established and function, is seen as a constraint, together with the public perception and values surrounding these areas. It is contended that the current state of protected areas is economically unsustainable and that these areas are expensive and should thus only represent a “small fraction” of the national conservation estate (Clements et al. 2022). This highlights a key risk facing South Africa’s protected area system as identified by Alberts et al. (2022), namely the manner in which we value protected areas and the expectations for these areas to “pay their way.”

The inclusion of ‘conservation areas’ within the White Paper further highlights the shift towards new conservation at a policy level. These areas are recognised as being similar to protected areas in terms of conservation objectives, however, they are not hindered by the numerous legislative restrictions placed on *inter alia* developments, access and multiple land use as is the case with formal protected areas (IUCN 2019). This inclusion aligns with the strategic direction of the CBD (1992), specifically the Kumming-Montreal Global Biodiversity Framework (Convention on Biological Diversity 2022) (GBF), which places an increased emphasis on effective area-based conservation measures and conservation outside of formally protected areas in pursuit of sustainable development and specifically nature’s contribution to people (Convention on Biological Diversity 2022: 1).

The implications for South Africa’s protected area to deliver on socio-economic development objectives are that these expectations will trickle down into protected area management plans with resultant repercussions. This may create potential uncertainty as to the purpose and objectives of the protected areas, which have been established with different objectives and are now being expected to reinvent themselves to deliver on others. Core questions which could arise include “What is the primary aim or objective of protected areas?”, sparking once again the debate around the divergent views of conservation and placing the country back at the very root of the problem which the new policy wishes to address.

Box 5: Four Policy Goals **Goal 1:** Enhanced biodiversity conservation

**Goal 2:** Sustainable use

**Goal 3:** Equitable access and benefit sharing

**Goal 4:** Transformed biodiversity conservation and sustainable use

## Objective 3: Possible Implications for Existing Protected Areas

It is evident from the above discussion that South Africa’s conservation policy affecting protected areas, although broadly new conservation focused, embodies and incorporates thinking aligned with all the four orientations. This suggests the potential for what Smith (1973) refers to as policy tensions created between the newly adopted ‘new conservation’ orientation and the policy implementation instruments, namely protected areas, which are predominantly aligned with traditional conservation. The implications of this should not be overlooked, especially against the statement in the White Paper that its aim is to provide “*policy certainty and a stable base for conservation, growth and sustainable development*” (RSA 2023: 26). The fact that all four orientations are embodied in the policy to a greater or lesser extent, however, serves to potentially create policy uncertainty and incoherence, hampering implementation. This is especially true when multi-directional “ambitious” and “sweeping” policy is drafted in the developing country context (Smith 1973). Within the context of South Africa, the new policy direction invokes a marked shift away from the traditional orientation. The result being that the current protected areas, largely founded and managed on the traditional conservation orientation and objectives, are expected to deliver on outcomes aligned with new conservation thinking. This tension is illustrated in Fig. 3 below. It is, thus, to be expected that in order to align with the new conservation policy orientation, existing protected areas will have to shift their current objectives away from those centred on traditional conservation. The inevitable result being a marked change in the manner in which these areas are managed in relation to aspects such as access, monetisation and use of resources.

Since the inception of the White Paper, several legislative developments in pursuit of the new conservation orientation have been effected in South Africa. Two developments in particular highlight this, namely the published draft National Biodiversity Economy Strategy (NBES) (RSA 2024b) and the draft notice calling for the exclusion of Environmental Impact Assessment (EIA) for development in the country’s iconic Kruger National Park (RSA 2024a; Patterson et al. 2024). The NBES was developed to optimise biodiversity-based business potentials within South Africa and to contribute to *inter alia* economic growth, poverty alleviation, local beneficiation and food security whilst purportedly maintaining ecological integrity of the biodiversity resource base (RSA 2024b: 6). The strategy has been developed to respond specifically to the White Paper, and acknowledges that it is explicitly about sustainable use (RSA 2024b: 6) in pursuit of delivering on the goals of the White Paper on the Conservation and Sustainable Use of South Africa’s Biodiversity.

The exclusion of EIA from the Kruger National Park is potentially another indicator of government’s pursuit of the new conservation orientation. Although not clear as to why EIA should not be utilised for developments in the park (Groundup 2024), it is easily argued that the instrument may be seen as a barrier to development within the protected area, and thus a hindrance to the achievement of the NBES, which calls for an expansion of ecotourism in the country’s protected areas and, specifically, in Kruger (see particularly Goal 1in the NBES) (RSA 2024b).

Given the adoption of the new policy, it is expected that the NEMPAA will be amended, with specific focus on the objectives of protected areas (Mokgohloa 2023). Such amendments will no doubt be brought about to align the act, and specifically protected area objectives, with the overarching new conservation orientation as set put in the overarching policy.

Given the above developments, and the fact that the policy orientation is clearly aligned with new conservation thinking, the resultant implications on South Africa’s existing protected areas of the policy shift as demonstrated by the conceptual framework, will possibly be:

- *Increased Monetisation*: Increased resistance to sustaining protected areas that fail to meet the economic expectations as espoused by the ‘new conservation’ orientation (See Clements et al. 2022). This could lead to defunding and de-proclamation of formal protected areas in pursuit of an increased focus on areas and landscapes and species most useful to humans in the form of socio-economic benefits (See Mascia and Pailler 2011, Qin et al. 2019). Arguably, it is protected area protected downgrading, downsizing, and degazettement (PADDD) as a result of socio-economic pursuits in lieu of conservation benefits which may threaten protected areas in southern Africa more specifically (See specifically Alberts et al. 2022 and Blackmore 2022).
- *Increased Access*: Increased support and pressure for development and land uses within, and adjacent to, protected areas that promote socio-economic development, but which are incompatible with historic traditional conservation objectives (See De La Fuente et al. 2020; Calderón et al. 2022 and Cilliers et al. 2024). Furthermore, adjacent communities will increasingly expect tangible benefits from protected areas through inter alia access to these areas and the resources therein.
- *Increased emphasis on utilitarian value*: Increased reliance on protected areas to provide resources or overall ecosystem services, potentially at the expense of species conservation. This could lead to an uncertain future for specific species and areas that do not offer tangible socio-economic benefits or demonstrable ecosystem services to society (Hauptfleisch et al. 2024).

## Conclusions

In this paper we have developed a conceptual framework that distinguishes the different prevailing conservation policy orientations. Its ability to separate these orientations has been illustrated through their relative positioning with the framework based on three axes encompassing spectra related to monetisation, intrinsic value, and access. When applied to the case of South Africa, the framework suggests a mismatch between the policy orientations of the existing protected areas mandate, and the foreseen goals of protected areas in the new conservation White Paper as arguably influenced by the CBD and the contemporary conservation discourse and thinking. Indeed, it is possible to establish a direction of travel which seems to suggest that the overarching policy direction is towards new conservation. Recent legislative developments in support of the White Paper support this. Although it is not appropriate for the researchers to judge which policy orientation should be followed, we have discussed the possible implications for the country’s system of protected areas. We recognise also that the identified implications will have differing operational implementation across the different types of protected areas. These operational implications may form the basis for future research. That being said, the mismatch in orientation between the overarching policy and the protected area system is illustrated. We suggest that the developed framework has potential for application to any conservation policies and can potentially act as a test as to the level of alignment between policy and policy implantation instruments.

## Supplementary information

Supplementary Information

## Footnotes

1 Within this context, two definitions are of importance, namely “protected areas” and “conservation areas” Protected Areas are defined as: *A Geographically defined area, declared or designated under NEM: PAA, which is designated, regulated and managed to achieve specific conservation objectives*. Conservation Areas are defined as: *Areas that are not protected areas but are managed in line with conservation principles*. For purposes of this paper, text dealing directly with protected areas was identified and analysed as “conservation areas” are not yet recognised within the legislative framework applicable to protected areas.
