# Supplementary material for: What Future for Protected Areas? Analysing the Mismatch between South Africa’s Pre-existing Protected areas System and the Declared vision in Contemporary Conservation Policy
Source: Environ Manage. 2024 Sep 23;74(6):1274–86. doi: 10.1007/s00267-024-02051-8 (PMC11549172; doi:10.1007/s00267-024-02051-8)
Supplement: Supplementary file 1 — Supplementary Information [file 267_2024_2051_MOESM1_ESM.docx]

**Table 1: Analysis of relevant policy provisions against the policy orientations**

| Policy Goals | **Protected Areas Relevant Policy Objective** | **Policy Orientation** | **Expected Policy Output** | **Policy Orientation** | **Expected Policy Outcome** | **Policy Orientation** |
| --- | --- | --- | --- | --- | --- | --- |
| **Goal 1: Enhanced biodiversity conservation: All biological diversity and its components conserved** | 1.1 Expand a representative system of **protected and conservation areas** that are effectively and efficiently managed | Traditional Conservation | A national cooperative programme, and prioritised plan of action, identifies terrestrial, freshwater, marine and coastal areas that support land- and seascapes, ecosystems, habitats, species and populations which contribute, or could contribute, to South Africa’s system of representative **protected and conservation areas** | Traditional Conservation | Expanded, connected, thriving, representative, inclusive and effectively managed **protected and conservation areas**, through co-ordinated partnerships  Contributions to global biodiversity **conservation** targets. | Traditional conservation  New Conservation |
|  |  |  | Connected, new, or extended **protected and conservation areas**, including through creating larger contiguous areas, as a means of improving the representation of terrestrial and marine ecosystem types, thereby enabling meeting national and international targets. | Traditional Conservation |  |  |
|  |  |  | Improved governance of state **protected areas.** | No specific orientation |  |  |
|  |  |  | Species of special concern identified, with viable populations effectively managed and protected, including within **conservation and protected areas.** | Traditional Conservation |  |  |
|  |  |  | Effective participation of land owners, businesses, traditional leaders, local communities and other interested and affected parties in expansion and custodianship of **protected and conservation areas.** | New conservation |  |  |
|  |  |  | Measures established to incorporate appropriate agro-ecosystems, agro-ecosystem management, agro-ecosystems authorisations, and **Protected Agricultural Areas** into conservation planning for OECMs. | New conservation |  |  |
|  | 1.2 Better integrate **conservation areas** into broader ecological and social land- and seascapes**.** | New conservation | Strategies, guidelines, mechanisms and incentives integrate **protected areas** within the broader ecological and social land- and seascapes and encourage conservation in adjacent private and communal buffer zones | New Conservation | Increased conservation viability and connectivity of land adjacent to **protected and conservation areas**, with effective human-wildlife conflict mitigation, and increased socio-economic development.  Conservation-compatible land use and sea use, and sustainable development, promote thriving land- and seascapes inside and outside **protected and conservation areas**. | New Conservation  Traditional Conservation |
|  |  |  | Biosphere reserves, natural world heritage sites, conservation land- and seascape initiatives, and conservation agriculture, entrench conservation land use and sea use outside of **protected areas.** | New Conservation |  |  |
|  |  |  | Activities in buffer zones adjacent to **protected and conservation areas** are compatible with and complement the area objectives | New Conservation  Traditional Conservation |  |  |
|  |  |  | Mitigation and/or conservation offsets from land transformation as a result of among others, mining and hard infrastructure, supports broader **conservation** outcomes. | New Conservation  Market Biocentrism |  |  |
| **Goal 4: Transformed biodiversity conservation and sustainable use:**  Effect is given to the environmental right as contained in Section 24 of the Constitution, which facilitates redress and promotes transformation. | 4.2 Position **protected and conservation areas** as catalysts of inclusive socio-economic development. | New Conservation | A strategy and action plan for state owned and other **protected areas** promotes economies of scale and enhance socio-economic outcomes. | New Conservation | Improved governance and management of **protected areas**, and contribution to the biodiversity economy, with meaningful community participation, influence and benefit from **protected areas**.  Protected and conservation areas provide access and benefit flows to communities, redressing past injustices, reducing disservices, and promoting support for **protected area and conservation area** persistence over alternative land uses. | New Conservation  Critical Social Science  Traditional Conservation |
|  |  |  | Institutional arrangements for state **protected areas** promote effective governance, and more equal and balanced partnerships with the private sector and communities. | New Conservation |  |  |
|  |  |  | Co-management arrangements with communities and arrangements for incorporation of community owned land are more equal, balanced and promote holistic outcomes | New Conservation |  |  |
|  |  |  | Biodiversity economy strategy promotes access and unlocks ecotourism and hunting benefit streams from protected areas to adjacent communities, with increased net benefit flows to people in and beyond **protected and conservation areas**. | New Conservation |  |  |
|  | 4.4 Promote participation and influence of designated groups (PDIs, youth, women, and people with disabilities) in **biodiversity conservation** and sustainable use. | New Conservation  Critical Social Science | A National Biodiversity Transformation Framework developed and implemented, with key interventions across the biodiversity sector that ensure:   - **Protected and conservation areas** are sustainably used for the benefit of people and nature, especially by communities surrounding **protected areas**. - Communities participate meaningfully in the full biodiversity based value chains from their communal land, as well as from adjacent **protected and conservation areas.** | New Conservation | The broad values of ecosystem services are realised and enhanced for designated groups. | New Conservation  Critical Social Science |

**Table 2: Analysis of existing protected areas against policy orientations**

| **Types of Protected Areas as set out in the National Environmental Management Protected Areas Act 57 of 2003** | **Objectives of PA declaration** | **Policy Orientation** |
| --- | --- | --- |
| **General purpose of protected areas**  **Section 17** | The purposes of the declaration of areas as protected areas are –  (a) to protect ecologically viable areas representative of South Africa’s biological diversity and its natural landscapes and seascapes in a system of protected areas; | Traditional Conservation |
|  | (b) to preserve the ecological integrity of those areas; | Traditional Conservation |
|  | (c) to conserve biodiversity in those areas; | Traditional Conservation |
|  | (d) to protect areas representative of all ecosystems, habitats and species naturally occurring in South Africa; | Traditional Conservation |
|  | (e)to protect South Africa’s threatened or rare species; | Traditional Conservation |
|  | (f) to protect an area which is vulnerable or ecologically sensitive; | Traditional Conservation |
|  | (g) to assist in ensuring the sustained supply of environmental goods and services; | New conservation |
|  | (h) to provide for the sustainable use of natural and biological resources; | New conservation |
|  | (i) to create or augment destinations for nature-based tourism; | Traditional Conservation  New conservation  Market Biocentrism |
|  | (j) to manage the interrelationship between natural environmental biodiversity, human settlement and economic development; | New conservation |
|  | (k) generally, to contribute to human, social, cultural, spiritual and economic development; or | New conservation |
|  | (l) to rehabilitate and restore degraded ecosystems and promote the recovery of endangered and vulnerable species. | Traditional Conservation |
| **Special Nature Reserves**  **Section 18(2)** | (a) to protect highly sensitive, outstanding ecosystems, species or geological or physical features in the area; and | Traditional Conservation |
|  | (b) to make the area primarily available for scientific research or environmental monitoring. | Traditional Conservation |
| **National Parks**  **Section 20(2)** | (a) protect— (i) the area if the area is of national or international biodiversity importance or is or contains a viable, representative sample of South Africa's natural systems, scenic areas or cultural heritage sites; or (ii) the ecological integrity of one or more ecosystems in the area; | Traditional Conservation |
|  | (b) prevent exploitation or occupation inconsistent with the protection of the ecological integrity of the area; | Traditional Conservation |
|  | (c) provide spiritual, scientific, educational, recreational and tourism opportunities which are environmentally compatible; and | Traditional Conservation |
|  | (d) contribute to economic development, where feasible. | New conservation |
| **National Park Wilderness Area**  **Section 22(2)** | (a) to protect and maintain the natural character of the environment, biodiversity, associated natural and cultural resources and the provision of environmental goods and services; | Traditional Conservation |
|  | (b) to provide outstanding opportunities for solitude; | Traditional Conservation |
|  | (c) to control access which, if allowed, may only be by non-mechanised means. | Traditional Conservation |
| **Nature Reserves**  **Section 23(2)** | (a) to supplement the system of national parks in South Africa; | Traditional Conservation |
|  | (b) to protect the area if the area – (i) has significant natural features or biodiversity; (ii) is of scientific, cultural, historical or archaeological interest; or (iii) is in need of long-term protection for the maintenance of its biodiversity or for the provision of environmental goods and services; | Traditional Conservation  New conservation |
|  | (c) to provide for a sustainable flow of natural products and services to meet the needs of a local community; | New conservation |
|  | (d) to enable the continuation of such traditional consumptive uses as are sustainable; or | New conservation |
|  | (e) to provide for nature-based recreation and tourism opportunities | Traditional Conservation |
| **Nature Reserve Wilderness Areas**  **Section 26(2)** | (a) to protect and maintain the natural character of the environment, biodiversity, associated natural and cultural resources and the provision of environmental goods and services; | Traditional Conservation |
|  | (b) to provide outstanding opportunities for solitude; | Traditional Conservation |
|  | (c) to control access which, if allowed, may only be by non-mechanised means | Traditional Conservation |
| **Protected Environment**  **Section 28(2)** | (a) to regulate the area as a buffer zone for the conservation and protection of a special nature reserve, national park, marine protected area, world heritage site or nature reserve; | Traditional Conservation |
|  | (b) to enable owners of land to take collective action to conserve biodiversity on their land and to seek legal recognition therefor; | New conservation |
|  | (c) to protect the area if the area is sensitive to development due to its – (i) biological diversity (ii) natural characteristics; (iii) scientific, cultural, historical, archaeological or geological value; (iv) scenic and landscape value; or (v) provision of environmental goods and services; | Traditional Conservation  New conservation |
|  | (d) to protect a specific ecosystem outside of a special nature reserve, national park, world heritage site or nature reserve; | Traditional Conservation |
|  | (e) to ensure that the use of natural resources in the area is sustainable; or | New conservation |
|  | (f) to control change in land use in the area if the area is earmarked for declaration as, or inclusion in, a national park or nature reserve. | New conservation |
